# Development and Validation of Quantitative Analysis Method for Phenanthrenes in Peels of the *Dioscorea* Genus

**DOI:** 10.4014/jmb.2206.06037

**Published:** 2022-07-26

**Authors:** Hunseong Kim, Thao Quyen Cao, Chae-eun Yeo, Seung Ho Shin, Hiyoung Kim, Dong-Hyuck Hong, Dongyup Hahn

**Affiliations:** 1 School of Food Science and Biotechnology, College of Agriculture and Life Sciences, Kyungpook National University, Daegu 41566, Republic of Korea; 2 Coastal Agriculture Research Institute, Kyungpook National University, Daegu 41566, Republic of Korea; 3 Department of Integrative Biotechnology, Kyungpook National University, Daegu 41566, Republic of Korea; 4 Department of Food and Nutrition, Institute of Agriculture and Life Science, Gyeongsang National University, Jinju 52828, Republic of Korea; 5 Department of Biomedical Science and Engineering, Konkuk University, Seoul 05029, Republic of Korea; 6 School of Bio-Industrial Machinery Engineering, Kyungpook National University, Daegu 41566, Republic of Korea

**Keywords:** *Dioscorea*, yam, HPLC, method validation, phenanthrenes, quantitative analysis

## Abstract

Phenanthrenes are bioactive phenolic compounds found in genus *Dioscorea*, in which they are distributed more in peel than in flesh. Recent studies on phenanthrenes from *Dioscorea* sp. peels have revealed the potential for valuable biomaterials. Herein, an analytical method using high-performance liquid chromatography (HPLC) for quantitation of bioactive phenanthrenes was developed and validated. The calibration curves were obtained using the phenanthrenes (1-3) previously isolated from *Dioscorea batatas* concentrations in the range of 0.625-20.00 μg/ml with a satisfactory coefficient of determination (R^2^) of 0.999. The limit of detection (LOD) and the limit of quantification (LOQ) values of the isolated phenanthrenes ranged from 0.78-0.89 and 2.38-2.71 μg/ml, respectively. The intraday and interday precision ranged from 0.25-7.58%. The recoveries of the isolated phenanthrenes were from 95 to 100% at concentrations of 1.25, 2.5, and 5.0 μg/ml. Additionally, phenanthrenes (1-3) were found in all investigated peel extracts. Hence, the developed method was encouraging for the quantitative analysis of phenanthrenes in genus *Dioscorea*.

## Introduction

Phenanthrenes are relatively rare, secondary metabolites derived through the formation of the oxidative coupling of the aromatic rings of stilbene precursors. More than 200 phenanthrenes have been isolated from plants belonging to the Annonaceae, Aristolochiaceae, Cannabaceae, Combretaceae, Cucurbitaceae, *Dioscorea*ceae, Euphorbiaceae, and Stemonaceae families [1, 2]. Phenanthrenes that have been isolated so far can be classified into three major groups by differences in their substitutents, which include monophenanthrenes, diphenanthrenes, and triphenanthrenes [3]. As of today, various biological activities of phenanthrenes have been reported, such as antimicrobial [3], antioxidant [4], anticancer [5], antiproliferative [6], anti-inflammatory [7], and antiallergic [8], and spasmolytic effects [3]. Moreover, phenanthrenes have been suggested to be a non-polar standard marker for genus *Dioscorea* [4].

The *Dioscorea* genus, widely known as yams, is the most important genus among the *Dioscorea*ceae family. The genus consists of over 600 species and is common in tropical and subtropical regions [9]. Yams have long been used as health food and folk medicine in some Asian countries due to their high nutritional value and various biological effects [9, 10]. The genus is regarded as an energy food source for consumers, especially in some local mountainous regions in Asia and Africa due to its high starch content, which can reach 80% on a dry weight basis [5, 11]. Reported pharmaceutical investigations showed that *Dioscorea bulbifera* ethanolic crude extract possesses a potent cytotoxic activity against human colorectal carcinoma (HCT116), human lung carcinoma (A549), and human breast carcinoma (MCF-7) [12]. The isolated compounds from *D. bulbifera* also displayed a potent effect on anti-HIV-1 integrase [13]. The anti-neuroinflammatory and neuroprotective activities of phenolic components isolated from *Dioscorea nipponica* were revealed in a recent study [14]. Phenanthrenes isolated from the *Dioscorea* genus reportedly possess anticancer [5], antioxidant [4], anticholinesterase [15], and anti-inflammatory activities [16]. Our previous studies also demonstrated anti-neuroinflammatory [17], antioxidant [4, 7], and inhibitory effects of phenanthrenes isolated from *Dioscorea batatas* peel on particulate matter-induced pulmonary injury [18]. Therefore, a valid quantitative analytical method for phenanthrenes is essential to evaluate the compositions of extracts from *Dioscorea* genus as a commercial medicinal herb or functional biomaterial source.

A recent study of ours revealed that the phenanthrenes content of *D. batatas* peel is markedly greater than that of its flesh [4]. Although Yoon *et al*. reported on the quantitative analysis method and its validation using high-performance liquid chromatography (HPLC) for phenanthrenes in roots of the *Dioscorea* genus [19], the method is limited in evaluating the different target analytes in the different matrix of yam peel. A dedicated analytical method was needed to simultaneously evaluate the exact amount of three bioactive phenanthrenes, which are correlated with bioactivity, in peels of the *Dioscorea* genus. In this investigation, we described the development and validation of a quantitative HPLC method using a photodiode array (PDA) detector to detect phenanthrenes in the peels of four *Dioscorea* species cultivated or collected in Korea, including *D. batatas*, *Dioscorea polystachya*, *Dioscorea quinqueloba*, and *D. bulbifera*. The results could provide a basis for quality assessment and standardization of phenanthrenes in the extracts of various *Dioscorea* species’ peels to discover further their full promise as functional biomaterials.

## Materials and Methods

### General Experimental Procedures

Acetonitrile, water, and methanol, all reagent grade, were purchased from J.T. Baker (USA). Trifluoroacetic acid (TFA) was purchased from Sigma-Aldrich (USA). Ethanol (extra-pure grade) was purchased from Duksan Pure Chemicals Co., Korea). Waters Alliance 2695 high-performance liquid chromatography (HPLC) (Waters, USA) which was performed on a Hector-M-C18 column (150 × 4.6 mm, 5 μm, RS Tech Corporation, Korea) was used for the analysis and the samples were detected by photodiode array detector (PDA, Waters 2996).

### Plant Material

*D. batatas* and *D. polystachya* tubers were purchased from Oneul-Achim (Korea). *D. bulbifera* was collected in Jeongeup, Korea, and *D. quinqueloba* was collected in Damyang, Korea. Their peels were separated from flesh and washed with water, then dried with a freeze-dryer (Ilshinbiobase, Korea).

### Preparation of Standard Compounds

Phenanthrenes, respectively named as 2,7-dihydroxy-4,6-dimethoxy phenanthrene (**1**), 6,7-dihydroxy-2,4-dimethoxy phenanthrene (**2**), and batatasin I (**3**), were isolated from the peel of *D. batatas* and identified following the previous description [4]. The purity of standard compounds was checked by HPLC. Their structures were confirmed by nuclear magnetic resonance (NMR) spectra and also through comparison with those reported in the literature.

### Sample Preparation

The freeze-dried peels of four *Dioscorea* species were powdered, and 1 g of dried powder from each sample was extracted with 20 ml of 95% ethanol for 2 h. Afterward, the samples were filtered with filter paper (Hyundai Micro CO., Korea) and evaporated in vacuo. The resultant extracts were weighed and dissolved in methanol to yield a concentration of 10 mg/ml. The extracts were then filtered by 0.45 μm syringe filters (Advantec, Japan) and injected into the HPLC system for analysis.

### Optimization of HPLC Analysis Conditions

The best analytical conditions were determined based on the methods suggested by Kim *et al*. [4] and Yoon *et al*.[19]. Parameters such as solvent, gradient composition, column temperature, and column type were evaluated to determine the optimal separation conditions for phenanthrenes from the *Dioscorea* peels. The mobile phases used were acetonitrile (**A**) and 0.1% TFA in water (**B**) at a flow rate of 1.0 ml/min. The gradient program was set as follows: 0-10 min, 20‒80% A; 10-12 min, 100% A; and 12-15 min, 20% A. The chromatograms were monitored at a wavelength of 261 nm, and the injection volume was 10 μl. The column temperature was maintained at 40°C throughout the analysis. The samples were injected 3 times each, and the averages of the peak areas on the chromatograms were obtained for quantitative analysis.

### Validation of the HPLC Method

The HPLC method for the determination of phenanthrenes (**1‒3**) was validated in terms of linearity, detection limit (LOD), quantitation limit (LOQ), precision, and accuracy [20]. The calibration curve for each phenanthrene was prepared at the six concentrations of 0.625, 1.25, 2.5, 5, 10, and 20 μg/ml. Calibration curves were obtained by averaging the peak areas of each compound on the chromatograms acquired from 3 injections that were monitored at a wavelength of 261 nm. The LOD and LOQ values of the developed method were calculated for phenanthrenes in the peels of *Dioscorea* extracts. The LOD and LOQ were calculated using the standard deviation (SD) and the slope (S) of the standard curve obtained and determined by regression analysis of the injection concentration and peak area using the following formula: [LOD =3.3*SD/S, LOQ =10*SD/S]. In addition, the intraday precision was calculated by five replicates of phenanthrenes at concentrations of 1.25‒10 μg/ml on the same day. The interday precision was assessed by analyzing phenanthrenes at the same concentrations (1.25‒10 μg/ml) on five different days. The precision was verified using the RSD value, which is the relative standard deviation of each result, following the formula: [RSD (%) = (SD/mean) * 100%]. The accuracy evaluation was measured by comparing the theoretical concentration (TC) and the actual concentration (EC) by injecting the spiked extracts. The spiking process was performed by mixing 250 μl of methanol (blank) and 2.5, 5, and 10 μg/ml standard mixture with 250 μl of 95% ethanol extraction of *D. batatas* peel at a low concentration. The percent recovery of each phenanthrene from the *D. batatas* extract was calculated as follows: [Recovery (%) = EC/TC × 100 (%)].

## Results and Discussion

**Fig. 1:**
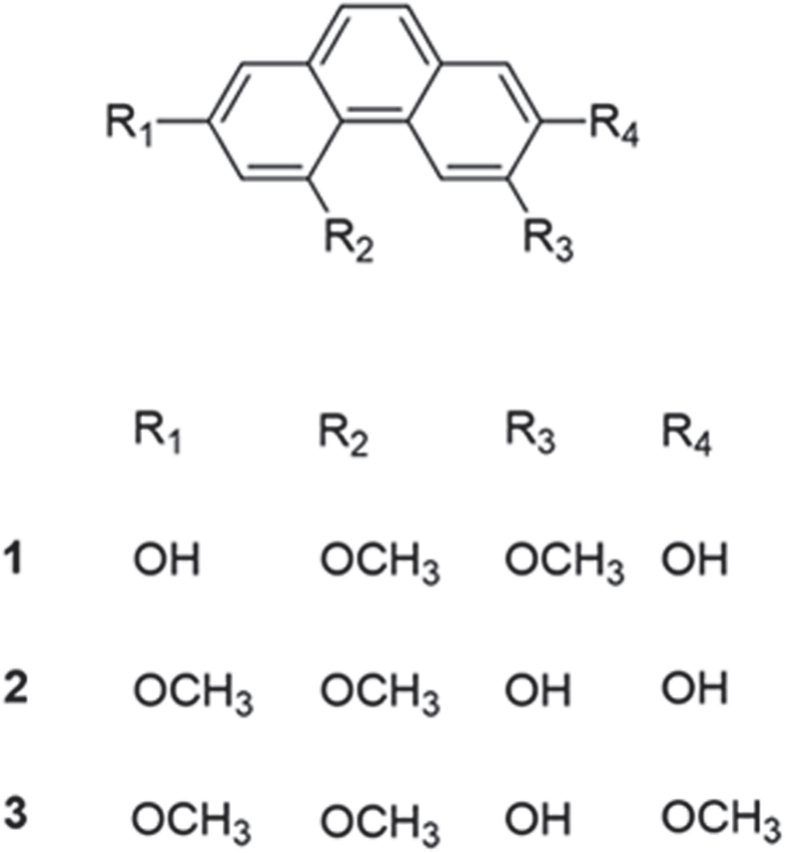
Chemical structures of phenanthrenes (**1‒3**) isolated from *D. batatas*.

**Fig. 2:**
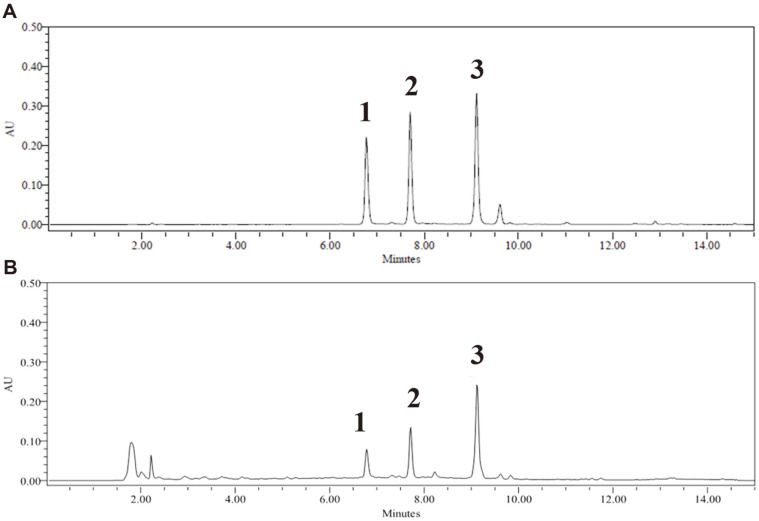
Chromatogram of phenanthrenes (**1‒3**) mixture (**A**) and 95% EtOH extract of the *D. batatas* peel monitored at 261 nm (**B**).

A method for quantitative analysis of three representative phenanthrenes (**1‒3**) (Fig. 1) in peels of four *Dioscorea* species using HPLC with a PDA detector was developed. The HPLC-PDA maximum absorbance wavelength of phenanthrenes was confirmed to be 261 nm. This method was modified from that of a previous report [4] by using different parameters to improve peak broadening and minimize running time and costs in routine analysis. From the chromatograms in Fig. 2, phenanthrenes (**1‒3**) were completely separated without overlapping with other peaks, and their retention times (r.t.) were 6.76, 7.69, and 9.10 min, respectively, shorter than those in the previous report [4]. In our study, linearity, the LOD, the LOQ, precision, and accuracy were validated [20, 21]. For the calibration curve preparation, standard solutions were prepared by serial dilution to the appropriate concentrations. As shown in Table 1, the linearity of calibration curves obtained from standard solutions was satisfactory with the determination coefficients (R^2^), which ranged from 0.9995 to 0.9996 in the concentration range of 0.625‒20.00 μg/ml. The LODs of phenanthrenes (**1‒3**) were 0.78, 0.82, and 0.89 μg/ml, respectively, and the LOQs were 2.38, 2.49, and 2.71 μg/ml, respectively (Table 1). Thus, the sensitivity of the HPLC-PDA method was determined to be appropriate for the quantitative detection of phenanthrenes in the *Dioscorea* genus.

In addition, accuracy and precision are the most important validation parameters in the assessment of an analytical method [22]. Precision and accuracy were analyzed using the relative standard deviation (RSD) and recovery, respectively. As the results show in Table 2, the precisions were 0.41‒1.55% and 1.66‒5.00% for phenanthrene **1**, 0.30‒1.31% and 2.39‒7.58% for phenanthrene **2**, and 0.25‒0.89% and 2.89‒5.58% for phenanthrene **3**, respectively. Furthermore, the recovery rates were 95.22‒100.80%, 95.07‒100.34%, and 95.87‒100.12% for phenanthrenes (**1‒3**), respectively (Table 3). The above results were acceptable to the method validation guideline presented by Association of Official Agricultural Chemists (AOAC) [23]. Hence, the HPLC-PDA method for phenanthrenes displayed good precision and accuracy at all concentrations in the *D. batatas* peel extract.

Species of the *Dioscorea* genus are known for their association with low-cost food culture, traditional medicine, modern Western medicine, and the pharmaceutical industry, as they possess various nutrients and phytochemicals, which differ in chemical structure, molecular weight, polarity, and other characteristics [9]. Across different geographic regions, diverse species of *Dioscorea* have been adopted within different habited areas as a food source due to the high nutritional benefits and therapeutic value for the treatment and cure of certain health problems [24‒26]. Previous extensive phytochemistry investigations carried out on the genus have resulted in the obtention of various bioactive metabolites such as allantoin [27], saponins [28], dioscorin [29], flavonoids [30], phenanthrenes [15‒19], and phenolic derivatives [31]. Phenanthrene-containing plants including the *Dioscorea* genus have been widely used as traditional medicines for the treatment of various diseases [1, 3]. To evaluate the content of phenanthrenes in the *Dioscorea* genus, the subsequent quantitative analysis with HPLC-PDA was investigated on the peel extracts of four *Dioscorea* species, including *D. batatas*, *D. polystachya*, *D. quinqueloba*, and *D. bulbifera*. Notably, all investigated *Dioscorea* species were found to possess antioxidant effects in a previous report [32]. In the present study, the results showed that phenanthrene **1** was the most abundant in the peel of *D. quinqueloba* with content of 173.69 μg/g on the basis of weight. The content of phenanthrene **2** was 166.99 μg/g based on weight, and was highest in the peel extract of *D. polystachya*. On the other hand, the peel extract of *D. polystachya* was also rich in phenanthrene **3** with content of 419.73 μg/g on the basis of weight. Therefore, phenanthrenes were most abundant in the peel of *D. polystachya* and least abundant in the *D. bulbifera* peel extract. Based on the results in Table 4, all standard phenanthrenes (**1‒3**) were presented in the peels of *D. batatas* and *D. polystachya*, and phenanthrenes (**2** and **3**) were not detected in the peel of *D. quinqueloba*, whereas phenanthrene 3 was not detected in the *D. bulbifera* peel extract.

In conclusion, phenanthrenes are a promising group of biologically active natural metabolites whose potential needs to be thoroughly investigated. Lots of phenanthrene-containing plants have been used in traditional medicine, including those of the *Dioscorea* genus. The proposed HPLC-PDA method was validated and developed for the quantification of phenanthrenes in the peels of *Dioscorea* spp. The method was optimized, and sample pretreatment was validated in terms of linearity, LOD, LOQ, precision, and accuracy. The obtained results showed that the developed method was suitable for the identification and quantification of phenanthrenes in the *Dioscorea* genus. Moreover, phenanthrenes (**1‒3**) were the most abundant in the peel extracts of *D. polystachya*. These findings suggest that more attention should be paid to investigating the bioactive phenanthrenes in the peel of *D. polystachya*.

## Figures and Tables

**Table 1:** Calibration, LODs, and LOQs of phenanthrenes (1‒3).

Compounds	Regression equation	Range (μg/ml)	R^2(a)^	LOD (μg/ml)	LOQ (μg/ml)
**1**	y = 87934x + 17518	0.625 – 20	0.9996	0.78	2.38
**2**	y = 109235x + 19608	0.625 – 20	0.9995	0.82	2.49
**3**	y=135505x + 28552	0.625 – 20	0.9995	0.89	2.71

^a^Coefficient of determination.

**Table 2:** Intraday and interday precisions of phenanthrenes (1‒3).

Compounds	TC^a^	EC^b^	RSD^c^
**1**	Intraday (*n* = 5)		
	1.25	1.18 ± 0.02	1.55
	2.5	2.51 ± 0.02	0.81
	5	5.11 ± 0.02	0.47
	10	9.97 ± 0.02	0.41
	Interday (*n* = 5)		
	1.25	1.14 ± 0.06	5.00
	2.5	2.48 ± 0.05	1.88
	5	4.98 ± 0.18	3.52
	10	9.85 ± 0.16	1.66
**2**	Intraday (*n* = 5)		
	1.25	1.18 ± 0.02	1.31
	2.5	2.52 ± 0.02	0.98
	5	5.09 ± 0.04	0.75
	10	9.97 ± 0.03	0.30
	Interday (*n* = 5)		
	1.25	1.10 ± 0.08	7.58
	2.5	2.46 ± 0.09	3.54
	5	4.92 ± 0.22	4.55
	10	9.77 ± 0.23	2.39
**3**	Intraday (*n* = 5)		
	1.25	1.20 ± 0.01	0.89
	2.5	2.51 ± 0.02	0.61
	5	5.09 ± 0.03	0.57
	10	9.97 ± 0.02	0.25
	Interday (*n* = 5)		
	1.25	1.24 ± 0.06	4.73
	2.5	2.64 ± 0.15	5.58
	5	5.22 ± 0.15	2.89
	10	10.31 ± 0.44	4.23

^a^Theoretical concentration (μg/ml); ^b^Experimental concentration (μg/ml); ^c^Relative standard deviation.

**Table 3:** Recovery of phenanthrenes (1‒3) with 95% ethanol extract of *D. batatas* peel.

ASCC^a^	TC^b^	EC^c^	RSD^d^	Recovery (%)
Compound **1**				
Blank	0.37	0.37 ± 0.00	0.98	
1.25	1.62	1.55 ± 0.01	0.90	95.22 ± 0.77
2.5	2.87	2.90 ± 0.03	0.95	100.80 ± 0.86
5	5.37	5.31 ± 0.06	1.19	98.77 ± 1.05
Compound **2**				
Blank	0.52	0.52 ± 0.01	2.02	
1.25	1.77	1.68 ± 0.01	0.46	95.07 ± 0.40
2.5	3.02	3.03 ± 0.04	1.25	100.34 ± 1.13
5	5.52	5.44 ± 0.06	1.18	98.60 ± 1.04
Compound **3**				
Blank	0.75	0.75 ± 0.02	2.11	
1.25	2.00	1.92 ± 0.01	0.44	95.87 ± 0.38
2.5	3.25	3.25 ± 0.04	1.32	100.12 ± 1.18
5	5.75	5.71 ± 0.08	1.41	99.31 ± 1.25

^a^Added standard compounds concentration; ^b^Theoretical concentration (μg/ml); ^c^Experimental concentration (μg/ml); ^d^Relative standard deviation.

**Table 4:** Phenanthrenes (1‒3) content in the *Dioscorea* genus extracts.

	Content (μg/g)				

		*D. batatas*	*D. polystachya*	*D. quinqueloba*	*D. bulbifera*
Compounds	**1**	77.35 ± 1.48	107.92 ± 2.99	173.69 ± 3.60	9.79 ± 0.65
	**2**	46.65 ± 1.14	166.99 ± 5.24	N.D.	4.24 ± 0.12
	**3**	97.19 ± 1.90	419.73 ± 12.02	N.D.	N.D.

N.D.: Non-detected.
